# Effect of Environmental Stress on the Nutrient Stoichiometry of the Clonal Plant *Phragmites australis* in Inland Riparian Wetlands of Northwest China

**DOI:** 10.3389/fpls.2021.705319

**Published:** 2021-08-19

**Authors:** Yi Zhou, Liang Jiao, Huijun Qin, Fang Li

**Affiliations:** ^1^College of Geography and Environment Science, Northwest Normal University, Lanzhou, China; ^2^Key Laboratory of Resource Environment and Sustainable Development of Oasis, Gansu, China

**Keywords:** inland riparian wetlands, clonal plant, stoichiometry, heterogeneous habitat, resource allocation trade-offs

## Abstract

Clonal plants play an important role in determining ecosystem properties such as community stability, species diversity and nutrient cycling. However, relatively little information is available about the stoichiometric characteristics of clonal plants and their drivers in inland riparian wetlands under strong environmental stress. In this manuscript, we studied the clonal plant *Phragmites australis* in an inland riparian wetland of Northwest China and compared its nutrient distribution and stoichiometry trade-offs as well as its responses to soil environmental factors in three different environments, namely, a wetland, a salt marsh, and a desert. We found that (1) *P. australis* could adapt to heterogeneous environments by changing its nutrient allocation strategies, as evidenced by the significant decrease in N and P concentrations, and significant increase in whole-plant C:P and N:P ratios from the wetland to the desert habitats. (2) *P. australis* adapted to stressful environments by changing its nutrient allocation patterns among different modules, showing a greater tendency to invest N and P in underground modules (rhizomes and roots) and an increase in the utilization efficiency of N and P in the leaves, and stems as environmental stress increased. (3) The C-N, C-P, and N:P-C in the whole plant and in each module showed significant anisotropic growth relationships in the three habitats (*P* < 0.05). (4) Soil water, pH and salt were the main factors limiting nutrient stoichiometry. The results of this study clarified the ecological adaptation mechanism of the clonal plant *P. australis* to heterogeneous environments and provided targeted protection strategies for inland riparian wetlands in Northwest China.

## Introduction

Ecological stoichiometry is the study of the distribution characteristics of elements within plants and the mechanisms of their interactions, which reflect the dependence, and demands of plants on natural resources (Elser et al., 2000; Güsewell, 2010; Gong et al., 2020). Carbon (C), nitrogen (N), and phosphorus (P) are the most important constituents of plants and play important roles in various physiological coordination processes, such as plant productivity, apoplastic decomposition, photosynthesis, genetics, and variation (Güsewell and Koerselman, 2002; Zhang H. et al., 2018). C provides the sugars necessary for plant growth, reproduction and structural development (Hessen et al., 2004; Elser et al., 2007; Yang et al., 2018), N plays a key role in controlling carbon assimilation and primary production (LeBauer and Treseder, 2008; Chen et al., 2016), P drives the production of genetic material, biofilms, and ribosomes (Westheimer, 1987; Agren, 2008; Dai et al., 2018; Huang et al., 2019). In particular, the stoichiometric C:N:P ratio has an important impact on ecosystem processes, such as material exchange, and energy cycling (Niklas et al., 2005; Yuan and Chen, 2009; Fan et al., 2015).

The accumulation and distribution of C, N, and P in plants also reflects the relationship between plants and the ecological environment (Minden and Kleyer, 2014; Wang et al., 2017; Shang et al., 2018). To improve our understanding of terrestrial ecosystems, the stoichiometric patterns and drivers of plant C, N, and P have been extensively studied in recent years in terms of the distribution patterns of C, N, and P in plant organs, and their relationships with environmental factors at global or regional scales (Sardans et al., 2017). For example, the biogeographic patterns of plant leaf N and P ratios differed at the global scale according to results from 1,280 plant species at 452 sample sites, and the concentrations of nitrogen and phosphorus in the leaves of terrestrial plants were related to latitude and the annual average temperature according to results from 753 species in China (Reich and Oleksyn, 2004; Han et al., 2005). These studies have shed some light on the distribution patterns of C, N, and P in plants and their relationships to the environment. However, the interactions of and differences in the distribution of nutrient elements among different organs of plants are still unclear, and the nutrient partitioning strategies among plant organs in response to the environment remain unconfirmed. For example, stoichiometric differences were more pronounced in stems and roots than in leaves and reproductive organs in both woody, and herbaceous plants (Kerkhoff et al., 2006). Therefore, it is necessary to carry out studies on the distribution patterns of nutrient elements in different plant organs in order to reveal the mechanisms of the relationships among plant organs, nutrients and the environment.

Clonal plants, also known as asexual plants, can produce offspring with the same genotype as the parent plant through asexual reproduction under natural conditions (McIntire and Waterway, 2002; Dong et al., 2014). Clonal plants exhibit different functions from nonclonal plants in various aspects of their growth metabolism and reproduction (Martínková et al., 2020). Clonal plants have a wider range of adaptive strategies, with unique functions related to nutrient absorption and spatial expansion (Hartnett and Bazzaz, 1983; Schmid and Bazzaz, 1987; Lopp and Sammul, 2016; Gao et al., 2020). Currently, most studies on clonal plant adaptation have focused on clonal growth conformation and morphological plasticity (Li et al., 2018), clonal partitioning and integration (Slade and Hutchings, 1987; Yu et al., 2004; Si et al., 2020; Wang et al., 2021), foraging behavior, and adaptive responses (Harris and Varga, 2021). However, there is a paucity of studies on clonal plants adapting to the environment through stoichiometric responses. Moreover, only a few studies have provided results showing significant differences in responses at different spatiotemporal scales. It has been shown that the leaf N concentrations and N:P of clonal plants decrease with increasing mean annual temperature (Hu et al., 2017), and it was also found that leaf N and P increased significantly with increasing mean annual temperature (Ying et al., 2015). Therefore, the study of the stoichiometric characteristics of individual modules of clonal plants can provide empirical results regarding resource acquisition and allocation trade-offs in response to heterogeneous conditions.

Wetland ecosystems are known as the “lungs of the earth.” In particular, inland riparian wetlands play a critical role in maintaining the structure, function, and hydrological cycles of oasis ecosystems in arid regions (Jiang and Xu, 2019). In recent years, inland riparian wetlands have become severely degraded due to disturbances from human activities and regional climate change *Phragmites australis* is a rhizomatous clonal plant that plays an important role in maintaining the stability, and function of inland riparian wetland ecosystems by adapting to different habitats due to its superb resistance to adversity. Therefore, we studied the clonal plant *P. australis* in the Dunhuang Yangguan National Nature Reserve wetland along an environmental gradient to address the following key scientific goals: (1) to clarify the stoichiometric characteristics of *P. australis* under stress conditions by comparing the spatial variations in *P. australis* stoichiometry among different habitats; (2) to reveal the nutrient allocation patterns of different plant modules by analyzing the anisotropic growth relationships in each *P. australis* module in different habitats; and (3) to clarify the main drivers of resource allocation trade-offs by studying the influence of environmental habitat factors on *P. australis* stoichiometry.

## Materials and Methods

### Study Site

**FIGURE 1:**
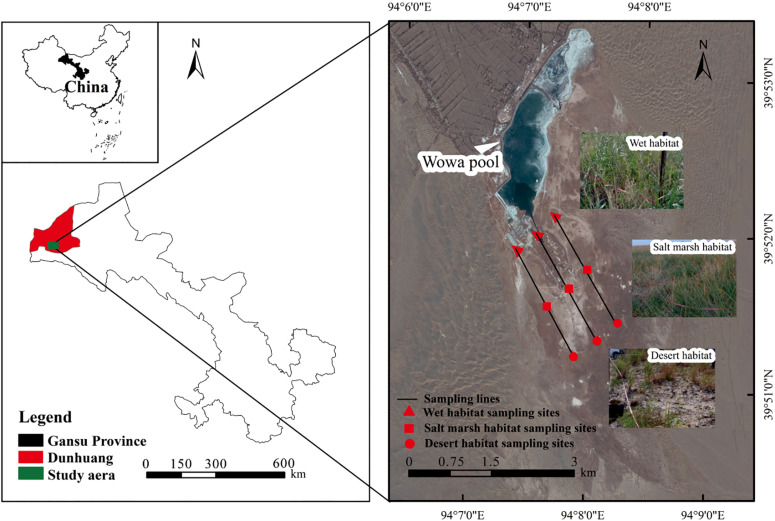
Study area and sampling point distribution.

The study area is located in the Yangguan National Nature Reserve of Dunhuang City in China (Figure 1), with a total area of 8.82 × 104 hm^2^ (39°39′–40°05′ N, 93°53′–94°17′ E, elevation between 1150 and 1500 m). The climate is a typical continental arid climate with large daily temperature differences, an annual mean temperature of 9.3°C, annual total precipitation of 36.9 mm, and annual evaporation of 2465 mm. The soil types are mainly meadow soil, marsh soil, and saline soil. The vegetation is mainly *P. australis*, as well as *Salicornia salsa*, *Lycium ruthenicum*, *Scorzonera austriaca*, *Glaux maritima*, and others.

### Experimental Design and Measurements

In September 2020, three parallel inside-out sampling transect were set up in Dunhuang Yangguan National Nature Reserve along the direction from wetland to desert, and were classified based on the distance from the reservoir (wetland: 500 m, salt marsh: 1500 m, and desert: 2500 m) and the density, cover, height, and frequency of *P. australis* (Table 1). Three 5 m × 5 m sample plots were randomly set up on three different habitat samples on each sampling transect, and the environmental factors such as elevation, latitude and longitude, and geographical topography were recorded for each sample plot.

**TABLE 1:** Characteristics of *P. australis* populations in the different habitats.

Habitat	Coverage (%)	Density (plant/m^2^)	Frequency (%)	Height (cm)	Biomass (g/m^2^)
Wetland	84.2 ± 6.36a	94.3 ± 7.65a	100.00 a	137.6 ± 7.25a	2047.95 ± 15.23a
Salt marsh	58.8 ± 6.58b	37.9 ± 10.4b	70.3 ± 4.48b	74.5 ± 4.84b	424.20 ± 15.77b
Desert	24.2 ± 1.67c	8.40 ± 3.93c	40.7 ± 6.72c	22.49 ± 2.84c	35.55 ± 7.78c

*Different lowercase letters from same column indicate significant differences between habitat variations (p < 0.05).*

Based on the characteristics of the clonal plant community, three whole *P. australis* plants with identical growth conditions were randomly selected from each sample plot of the same habitat. All aboveground and underground parts of the selected plants were completely harvested according to the clonal module collection method of “tracking and digging” based on the direction of the rhizomes in each plot (Harper, 1977; Dong et al., 2011). To prevent enzyme activity from occurring in the samples, the samples were heated at 105°C for 30 min. Then, all *P. australis* plants were separated into their leaves, stems, roots, and rhizomes. All samples were numbered and dried to constant weight at 80°C. The dried plant samples were ground using a hybrid ball mill (MM400, Retsch, Germany) and passed through a 100 mesh sieve.

The soil water concentration was determined by the oven drying method, the soil bulk weight was measured by the ring knife method, the soil pH was determined by the PHS-SD pH meter (Beijing Tongde Venture Technology Co., Ltd., Beijing, China), and the soil salt concentration was determined by the electrical conductivity method.

Samples of *P. australis* (leaves, stems, rhizomes, and roots) were weighed out to 0.1 g each. The C concentration of *P. australis* was measured using wet oxidation with the Walkley–Black K_2_Cr_2_O_7_-H_2_SO_4_ oxidation method (Nelson and Sommer, 1982). Samples of *P. australis* (leafs, stems, rhizomes, and roots) were weighed to 0.3 g each. The N and P concentrations were determined by an automated chemical analyzer after adding H_2_SO_4_-H_2_O_2_ to prevent boiling (Smartchem 200, Advanced Monolithic Systems, Graz, Italy).

### Data Analysis

The significance of the differences in nutrient stoichiometry and soil characteristics in the different habitats were tested by one-way ANOVA, and LSD. The relationship was analyzed by calculating Pearson correlation coefficients between the stoichiometry and population characteristics of *P. australis*. The above methods were implemented in SPSS 22.0 (SPSS Inc., Chicago, IL, United States).

Standardized major axis (SMA) analysis was implemented by the “smatr” package in R 3.6.1 (R Core Team, 2015) to calculate the anisotropic growth indices and constants of C, N, and P, and their ratios in *P. australis* in different habitats. The calculation formula was as follows:

(1) logy=blog⁡x+log⁡a

where, *y* represents the dependent variable and *x* represents the independent variable. In this study, *x* and *y* represent the C, N, and P concentrations of the whole *P. australis* plant and its individual modules, *a* is the anisotropic constant indicating the intercept of the linear function, and *b* is the allometric growth index showing the slope of the linear function. When *b* = 1, it means that the dependent variable and the independent variable are in an isokinetic growth relationship; when *b* > 1 or *b* < 1, the two have an the allometric growth relationship (Gargaglione et al., 2010).

Redundancy analysis (RDA) was applied in CANOCO to analyze the response relationships of C, N, and P and their ratios in each organ of *P. australis* to soil environmental factors (water, salt, pH, and bulk density), and we also made a RDA with all the data to explore the driving forces of *P. australis* stoichiometry. Before the RDA, detrended correspondence analysis was performed on the dataset to ensure that the gradient lengths were consistent with those in the linear model. All data were log-transformed before parameter testing and passed the *F*-test.

A structural equation model (SEM) was applied to evaluate and quantify the effects of soil physicochemical factors on whole-plant C, N, and P, and their ratios. Before modeling, we examined the distributions of all variables and checked their normality. To satisfy the assumption of a normal distribution, specific values of certain soil factors as well as the whole-plant C, N, and P, and their ratios were ln transformed to improve the normality of the distribution. After data processing, we tested the overall goodness-of-fit of the model. The model chi-square test *p*-values were greater than 0.05, RMSEA < 0.05, *P* > 0.05, and both GFI and AGIF > 0.90, indicating that the model fit was good. The SEM was implemented *via* the R “lavvan” package and visualized *via* the “semPolt” package.

## Results

### Characteristics of Soil Environmental Factors in the Different Habitats

**FIGURE 2:**
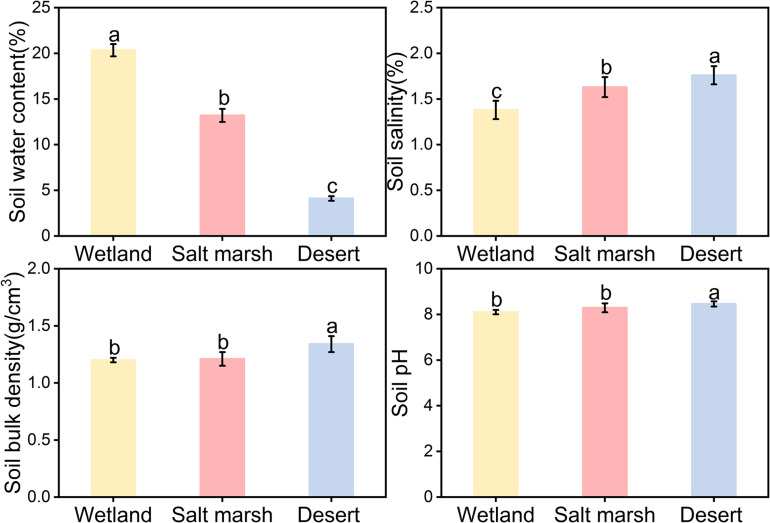
Characteristics of soil factors under different habitats (mean ± SE, different lowercase letters indicate significant differences between habitat gradients).

Figure 2 shows that the average soil water concentration showed a gradual decreasing trend from wetland to desert habitats, i.e., wet (20.34 ± 0.67%) > salt marsh (13.20 ± 0.72%) > desert (4.11 ± 0.25%). Soil salt, soil bulk density, and soil pH showed gradual increasing trends, i.e., desert habitat (salt 1.76 ± 0.10%, bulk density 1.34 ± 0.02 g/cm^3^, and pH 8.46 ± 0.19) > salt marsh habitat (salt 1.63 ± 0.59%, bulk density 1.21 ± 0.15 g/cm^3^, and pH 8.29 ± 0.03) > wetland habitat (salt 1.38 ± 0.52%, bulk density 1.34 ± 0.11 g/cm^3^, and pH 8.10 ± 0.10).

### Characteristics of *P. australis* Stoichiometry in the Different Habitats

**FIGURE 3:**
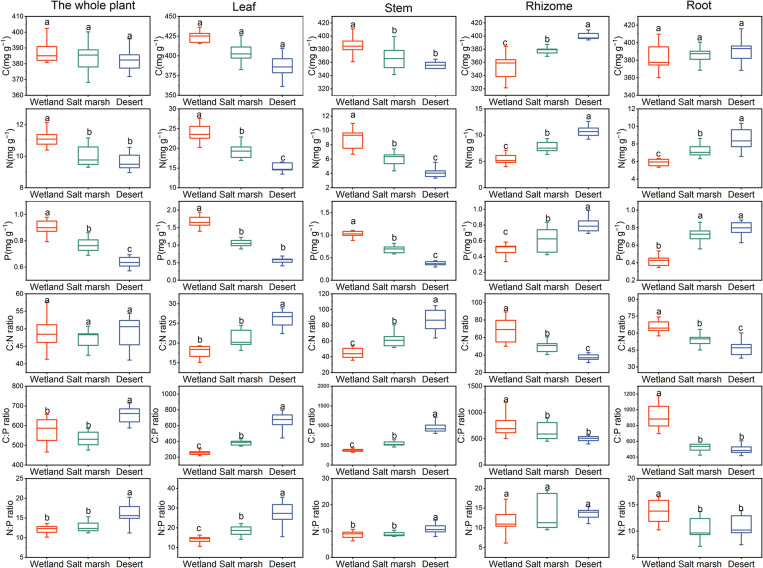
The stoichiometry of *P. australis* under different habitats (means ± se, different lowercase letters indicate significant differences between habitat gradients).

Figure 3 shows that the C concentration of the whole plant, leaves and roots were not significantly different from the wetland to desert habitats (*P* > 0.05), while that of rhizomes increased significantly, and with wetland habitat (355.27 ± 6.82 mg/g) < salt marsh habitat (378.08 ± 2.18 mg/g) < desert habitat (400.28 ± 1.83 mg/g). The C concentration in the stems decreased significantly between the wetland and salt marsh habitats (*P* < 0.05). The harsher the environment was, the less N and P concentrations there was in leaves and stems, and the more N and P was received by roots and rhizomes. In addition, the C:N in the whole plant did not change significantly from wetland to desert habitats (*P* > 0.05), while the C:N in leaves and stems showed a significant increase, i.e., wetland (leaves 17.58 ± 0.29 mg/g and stems 44.29 ± 2.20 mg/g) < salt marsh (leaves 20.25 ± 0.99 mg/g and stem 62.93 ± 3.56 mg/g) < desert (leaf 25.58 ± 0.37 mg/g and stem 86.27 ± 5.15 mg/g). The C:P ratio in whole plants, leaves and stems increased with increasing environmental stress, while the C:P ratio in roots (502.39 ± 20.17 mg/g), and rhizomes (500.37 ± 18.66 mg/g) was the lowest in the desert habitat. The N:P ratio in whole plants, leaves and stems increased significantly with environmental stress (*P* < 0.05).

**FIGURE 4:**
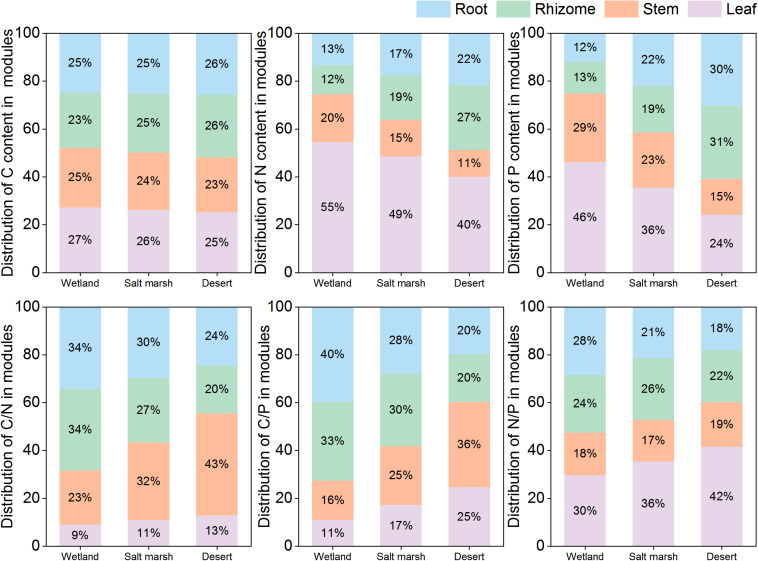
Modules allocation of stoichiometry of *P. australis* in different habitats.

With environmental stress, the C concentration in each module did not change significantly, but that in leaves was higher than those in the other modules (Figure 4). The proportions of N and P in leaves and stems gradually decreased from the wetland to desert habitats; in contrast, the proportions of N and P in roots, and rhizomes gradually increased. The C:N and C:P ratios in stems and leaves and the N:P ratio in leaves gradually increased from the wetland to desert habitats, and while the N:P ratio in roots gradually decreased.

### Allometric Growth Relationships Indicated by the Stoichiometry of *P. australis* in Different Habitats

In the whole plant, the C-N, C-P and N:P-C ratios showed significant anisotropic growth relationships in all three habitats, but the anisotropic index differed among habitats, and the maximum anisotropic index of C-N and C-P was reached in the salt marsh habitat (Supplementary Table 1). In leaves, the C-N ratio showed significant anisotropic growth relationships in the wetland and salt marsh habitat. The C-P ratio showed significant anisotropic growth relationships from the wetland to the desert, and the anisotropic growth index first increased and then decreased (Supplementary Table 2). In stems, the C-N, C-P, and N:P-C ratios showed significant anisotropic growth relationships in all three habitats, and the maximum anisotropic index for N:P-C was reached in the salt marsh habitat (Supplementary Table 3). In the rhizome modules, the C-N, C-P, and N:P-C ratios showed significant anisotropic growth relationships, with the anisotropic index for rhizome C-N increasing with increasing environmental stress (Supplementary Table 4). In the roots, the C-N, C-P, and N:P-C ratios showed significant anisotropic growth relationships in all three habitats, and the anisotropic indices of C-N and C-P increased with increasing environmental stress (Supplementary Table 5).

### Relationship Between Stoichiometry and Population Characteristics of *P. australis*

The coverage, density, frequency, height and biomass of *P. australis* showed significant positive correlations with N and P concentration, and significant negative correlations with N:P (*P* < 0.01, Table 2). Meanwhile, the coverage, frequency, and height of *P. australis* showed significant negative correlations with C:P (*P* < 0.05). However, there was no significant correlation between C, C:P and coverage, density, frequency, and height (*P* < 0.05).

**TABLE 2:** Correlation between stoichiometry and population characteristics of *P. australis*.

Correlation coefficient	C	N	P	C:N	C:P	N:P
Coverage (%)	0.261	0.664**	0.903**	−0.520**	–0.007	−0.707**
Density (plant/m^2^)	0.272	0.697**	0.896**	–0.370	0.057	−0.626**
Frequency (%)	0.267	0.681**	0.908**	−0.473*	0.015	−0.685**
Height (cm)	0.269	0.688**	0.907**	−0.443*	0.028	−0.670**
Biomass (g/m^2^)	0.270	0.693**	0.861**	–0.266	0.094	−0.556**

*Significant at the 95% confidence level. **Significant at the 99% confidence level.

### Soil Factors Influencing of Stoichiometry of *P. australis* in Different Habitats

**FIGURE 5:**
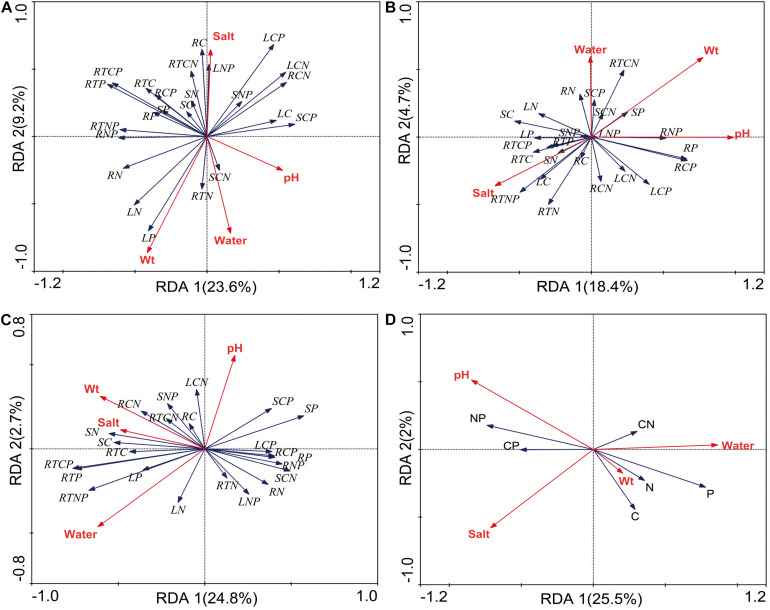
Redundancy analysis (RDA) of the relationship between the *P. australis* stoichiometry and soil factors **(A)** wetland, **(B)** salt marsh, **(C)** desert, and **(D)** all habitats; Wt represent bulk density; L represent leaf, S represent stem, R represent rhizome, and RT represent root.

The relationships between stoichiometry and soil physicochemical properties were deemed significant based on the RDA, which confirmed that soil water, salt, pH, and bulk were the main forces driving resource allocation in *P. australis* (Figure 5). In the wetland habitat, the soil bulk density explained a substantial amount of the variation in plant stoichiometric characteristics and had a significant negative correlation with C and N in roots (*P* < 0.05). In the salt marsh habitat, the soil pH and salt concentration were important driving forces; the N concentration and N:P ratio in roots had significant positive correlations with soil salt, and the P concentration and N:P and C:P ratios in rhizomes had significant positive correlations with soil pH (*P* < 0.05). In desert habitats, soil water was an important driving force; the C:P and N:P ratios in roots had significant positive correlations with soil water, and the C:P ratio and P concentration in stems showed negative correlations with soil water (*P* < 0.05). In all habitats, soil salt, pH and water can explain well the stoichiometry variability of *P. australis*. Soil salt and pH showed significant negative correlations with P concentration. Soil water showed significant positive correlations with N and P concentrations and negative correlation with N:P (*P* < 0.05).

**FIGURE 6:**
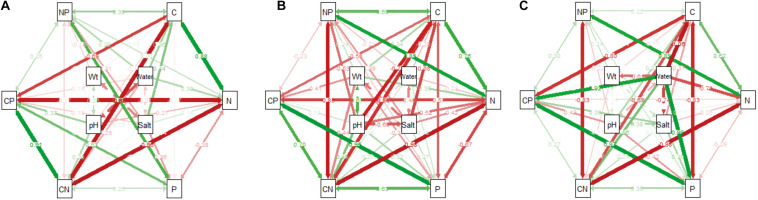
The influence pathways and flux of different habitats on the stoichiometry of *P. australis* by structural equation model analysis (SEM) **(A)** wetland, **(B)** salt marsh and **(C)** desert; CN represents the C:N ratio; CP represents the C:P ratio; and NP represents the N:P ratio.

In the wetland habitat, the SEMs showed that soil water, salt, bulk density, and pH had no effect on the stoichiometry of *P. australis* (*P* > 0.05). In the salt marsh habitat, soil salt, and pH were the most important factors affecting the stoichiometry of *P. australis*. The path coefficient from soil salt to C was –0.68 and those from soil pH to N and C:N were –0.52 and 0.48, respectively. In the desert habitat, soil water was the most important factor affecting the stoichiometry of *P. australis*, and the path coefficients of water to C, N, P, and C:P were –1.06, –0.70, 0.99, and 0.92, respectively (Figure 6).

## Discussion

### Characteristics of Resource Allocation Trade-Offs for the Clonal Plant *P. australis* in Heterogeneous Environments

In the course of long-term adaptive evolution, clonal plants have developed ecologically adaptive responses to effectively utilize heterogeneous resources, and resist various environmental stresses. The stoichiometry of *P. australis* is able to control its underlying physiological and biochemical processes in response to environmental changes, and reflecting a key survival strategy of clonal plants (Güsewell et al., 2003; Pinno and Wilson, 2014; Zhang J. H. et al., 2018). In this study, it was found that the C concentration of the whole plant did not change significantly, while the N and P concentrations showed a decreasing trend and the coverage, density, frequency, height, and biomasst of *P. australis* also showed a decreasing trend with increasing environmental stress (Figure 3 and Table 1). This suggests an ecological response of the whole plant to heterogeneous habitats under environmental stress conditions (Gong et al., 2018). Meanwhile, the coverage, density, frequency, height and biomass of *P. australis* showed significant positive correlations with N and P concentrations and significant negative correlations with N:P (*P* < 0.01, Table 2). This is due to changes in population characteristics that alter the source-sink relationships of nutrients in plants to adapt to the heterogeneous environmental resources and population competition (Ackerly, 2004; Amatangelo and Vitousek, 2008). There was no significant change in the proportion of C distributed within each module (Figure 4). This may have been due to the relatively stable concentration of C as the basic structural element of plant tissues (Yang et al., 2015). Meanwhile, the proportions of N and P in leaves were greater than those in other modules in all three habitats (Figure 4). The same results were obtained from studies on perennial herbaceous plant leaves on the northern Tibetan Plateau (Ma et al., 2019). The reason for these results is that leaves are the primary photosynthetic site in plants. Moreover, N and P are the main elements making up the enzymes required for photosynthesis and for the synthesis of genetic material (Bui and Henderson, 2013). From the wetland to desert habitats, the N and P concentrations in leaves and stems decreased significantly, while the proportions of N and P in the rhizomes increased (Figures 3, 4). The results of this study are consistent with the theory of optimal plant nutrient allocation, which suggests that plants achieve an optimal degree of nutritional coordination in different environments through the rational distribution of substances and energy (Vitousek, 1982). In particular, clonal plants invest more N and P in rhizomes and root modules to increase their asexual reproduction rate, and escape from patches with poor resource levels to patches with better conditions as environmental stress intensifies (Hutchings, 1994; Wang et al., 2008; Zhang et al., 2019b). The results of this study were consistent with those of other studies on the dominant plant communities in the desert areas of northern China (Yang et al., 2018). These changes may occur to increase plant root vigor in order to obtain more nutrients to adapt to and escape from harsh external environmental conditions (Cai et al., 2019). However, studies on Leymus chinensis in northeastern China have come to the opposite conclusion, showing that nutrient addition increased asexual reproduction (Li et al., 2021). This may be related to the different environmental contexts of the study areas. Our study area is located in an arid zone with a high degree of soil salinization and drought stress. To maintain the population, clonal plants have to escape from patches with poor resource levels to patches with better conditions through asexual reproduction.

The ratios between nutrients are an important reflection of the rapidity of the growth rate, the efficiency of nutrient utilization, and the limiting elements. They are also an effective indicator of environmental nutrient limitations and plant growth strategies (Vrede et al., 2004; Cao et al., 2020; Xu et al., 2020). The results of our study found no significant changes in C:N in the whole plant with increasing environmental stress (Figure 3). This is attributed to the greater stability of C:N in plants compared to C:P and N:P (Li et al., 2017). Meanwhile, the whole-plant C:P increased significantly from the salt marsh to the desert habitat (Figures 3, 4). This was due to the increase in P availability in the whole plant when the resources required by *P. australis* were under severe stress (Herbert et al., 2003). It has been suggested that plant N:P ratios can be used as indicators of nutrient limitation in soils; N:P < 14 is interpreted as N limitation, N:P > 16 as P limitation, and 14 < N:P < 16 as N and P colimitation (Koerselman and Meuleman, 1996). In this study, the mean N:P values of *P. australis* leaves in wetland, salt marsh and desert habitats were 16.50, 21.16 and 27.74, respectively, all of which are greater than 16, and indicate P limitation. The close proximity of the study region to the desert may mean that it is subjected to severe wind erosion, which leads to substantial losses of P from soil (Song et al., 2016). The N:P ratios in stems, roots and rhizomes were less than 14 in all three habitats, and the N:P ratios in stems and rhizomes increased with increasing environmental stress (Figures 3, 4). Water stress can reduce the activity of nitrate reductase and nitrate nitrogen uptake by roots by slowing nitrate reduction and ammonia assimilation, and causing nitrogen limitation in plants (Luo et al., 1994). Therefore, nutrient limitation in different modules may be an entry point for performing inland riparian wetland management and restoration. The C:N, C:P and N:P ratios in leaves increased significantly from the wetland to desert habitats (Figure 4). This result is consistent with studies of *Zygophyllum xanthoxylum* under different environmental conditions (Niu et al., 2018). The plants maintained higher C:N and C:P ratios in leaves to help improve their competitiveness in droughty and nutrient-poor environments (Zhang et al., 2017). The C:N ratios of roots and rhizomes showed a decreasing trend, and reflecting the transfer of nutrient elements from aboveground modules to belowground modules.

Anisotropic growth relationships are often used to reflect differences in the ability of plants to absorb, transfer, distribute, and utilize nutrients during growth. They also reflect the change patterns of two attributes in an organism along with its growth and development process (Zhang et al., 2019a). Anisotropic growth analysis has been widely used to study the size dependence of different plant structures and processes (Gould, 1966; Niklas et al., 2005; Zhang et al., 2021). It is also well suited for testing scale dependence in resource allocation and has been successfully applied to resource allocation studies (Klinkhamer and Meelis, 1990). The C-N, C-P, and N:P-C ratios in the whole plant as well as in the individual modules showed significant anisotropic growth relationships in all three habitats, demonstrating the similarity of the respective nutrient allocation patterns to different modules. However, the anisotropic growth indices of the whole plant and individual modules related to C-N, C-P, and N:P-C varied among the different habitats (Supplementary Tables 1–5). The stressful environment influenced the rates of change in nutritional element concentrations, and the analysis revealed differences in the rates of change in C, N, and P concentrations in the different habitats. The findings were similar to the relationship between C-N and C-P observed during the allometric growth of *Suaeda salsa* in different habitats in coastal wetlands (Liu et al., 2015). Meanwhile, N-P in leaves showed a significant anisotropic growth relationship in desert habitats. The same conclusion was obtained in arid environments in Northwest China, indicating that environmental stress altered the N-P anisotropic growth relationship in leaves (Wang et al., 2015). In addition, the C-P anisotropic growth index of both whole plants and aboveground modules reached a maximum in the salt marsh habitat. This might be related to the different C, N, and P allocation strategies in the different habitats.

### Drivers of Resource Allocation Trade-Offs for the Clonal Plant *P. australis* in Heterogeneous Environments

Plant growth and development are closely related to the habitat in which plants live, and changes in plant stoichiometry can reflect plant adaptations to different environmental conditions (Li et al., 2013; Yu et al., 2020). As the main source of nutrients for vegetation in an ecosystem, the soil environment plays an important role in determining community structure, and ecosystem stability (Sun et al., 2017).

In all habitats, soil salt, pH, and water were the main drivers of *P. australis* stoichiometry. Soil water showed significant positive correlation with N and P concentrations (*P* < 0.05, Figure 5). The same results were obtained from a study on *Reaumuria soongorica* stoichiometry in desert areas of China (Ma et al., 2008). This indicates that the increase in soil water can promote P uptake of *P. australis*. And plant may adapt to the water restriction by regulating the nutrient utilization strategies (Yang et al., 2016). Meanwhile, soil water had significant positive correlation with N and P concentrations and negative correlation with N:P based the RDA, and it also showed that the N and P concentrations of *P. australis* decreased with increasing soil salt and pH. The growth and development of *P. australis* are significantly restricted by soil salt and pH. It proved that *P. australis* can change the osmotic regulation level by adjusting the N and P concentrations to enhance the adaptability to extreme environments (Mansour, 2000).

In the wetland habitat, the SEM showed that soil water, salt, bulk density, and pH had no significant (*P* > 0.05) effect on the stoichiometry of *P. australis* (Figure 6). *P. australis* grows well with an appropriate combination of water, heat and ample nutrients in wetland habitats, and its requirements in terms of soil environmental factors were met in the wetland environment in this study. However, RDA showed that the soil bulk density had a significant (*P* < 0.05) negative correlation with C and N in roots of the *P. australis* modules (Figure 5). The number of plant roots may have decreased as the soil density increased, which would have affected the total C and P uptake by roots (Logsdon and Karlen, 2004).

In salt marsh habitats, changes in *P. australis* stoichiometry are the result of plant adaptive evolution driven by environmental stress and reflect the ability of the plant to adapt to adversity (Hong et al., 2015). The SEM showed that soil salt and pH were the most important drivers of whole-plant stoichiometry. Soil salt showed a negative correlation with the whole-plant C concentration, and the path coefficient was –0.68 l the path coefficients of soil pH for the whole-plant N and C:N were –0.52 and 0.48, respectively (Figure 6). These relationships were due to the severe salinization caused by the scarce precipitation, strong evaporation, and shallowly buried, poorly drained wetlands in the study area. Thus, changes in certain structures and functions might be induced in plants under saline stress in arid zones; these changes would in turn affect the physiological photosynthetic properties of plants, disrupt the ionic balance in plants, and disrupt protein synthesis, thereby reducing the whole-plant C and N concentrations (Qiang et al., 2018). Therefore, plants can enhance their adaptability to extreme environments by increasing their N utilization efficiency. However, studies on the relationship between *P. australis* and soil salt in the middle reaches of the Tarim River revealed no significant correlation (Thevs et al., 2007). This conclusion is contrary to our results, suggesting that the influences of environmental factors on plants differ at the regional scale. RDA showed that the N concentration and N:P ratio in roots exhibited a significant positive correlation with soil salt (Figure 5). The same results were obtained from a study on the roots of six typical desert plants in the Tarim Basin, which showed that high soil salt levels promote root N uptake (An et al., 2017). The P concentration and N:P ratio in rhizomes showed a significant positive correlation with soil pH (Figure 5). The availability of nutrients to the belowground modules of the clones can increase as the salinity of the soil increases, thus enabling the plants to escape from unfavorable habitat patches as soon as possible by spreading over long distances.

In desert habitats, plants face the dual stresses of water deficit and nutrient limitation. Therefore, their nutrient uptake or nutrient utilization capacity is a key mechanism by which plants adapt to arid habitats (He et al., 2015). The SEM showed that soil water was the most important driving force behind nutrient uptake, with water path coefficients of –1.06, –0.7, 0.99, and 0.92 for C, N, P, and C:P, respectively (Figure 6). Similar results were obtained for the changes in P concentration in plants from 155 drought-treated or persistent drought zones worldwide (He and Dijkstra, 2014). This can be attributed to the fact that water is the limiting factor in the arid zone and that the decrease in soil water concentration limits the availability of soil P, and reduces the amount of P available to plants. Soil water was negatively correlated with whole-plant C and N because a high soil water concentration accelerates plant biomass accumulation and has a diluting effect on N elements (Reich and Oleksyn, 2004). Meanwhile, RDA showed that soil water had a positive correlation with C:P and N:P in roots and a negative correlation with C:P, and P in stems (Figure 5). This indicates that the reduction in water increased the efficiency of P utilization by roots and stems. To improve resistance to drought and salt stress, the C:P ratio in stems and roots increases, the growth rate slows, and water consumption decreases accordingly. This finding conforms to the growth restriction hypothesis, which states that the plant growth rate is adjusted to adapt to changes in the external environment by adjusting the stoichiometric ratio of elements during plant growth, and development (Wu et al., 2021).

Global warming will further intensify in the future, and the degree of drought in the inland rivers of Northwest China will further intensify in duration, extent, and degree. In addition, clonal plants are very sensitive to global changes, and increasing drought conditions and unpredictable rainfall events will alter soil nutrient effectiveness, and thus affect plant nutrient stoichiometry. Therefore, clonal plants under different environmental conditions should be managed differently on the basis of their response characteristics.

## Conclusion

In this study, resource allocation trade-offs and their driving forces in the clonal plant *P. australis* in different habitats of inland riparian wetlands were explored at two scales: the whole plant and individual modules. As environmental stress increases, *P. australis* prefers to invest in underground modules for survival growth, and the whole plant responds to habitat changes by decreasing its N and P concentrations and increasing its C:P values. C-N, C-P, and N:P-C in the whole *P. australis* plant as well as in individual modules showed significant anisotropic growth relationships in all three habitats. In addition, soil water, salt, and pH were the most important environmental driving factors behind resource allocation trade-offs in *P. australis*. The conclusions of this study help to further characterize the nutrient requirements of clonal plants and the existence of mutual environmental feedbacks, which can be used to describe the ecological processes in wetlands from multiple perspectives and provide scientific guidance for the conservation, and development of wetlands.

## Data Availability Statement

The original contributions presented in the study are included in the article/Supplementary Material, further inquiries can be directed to the corresponding author.

## Author Contributions

LJ and YZ conceived and designed the study. YZ, FL, and HQ collected the data. YZ and LJ wrote, reviewed, and edited the manuscript. All authors read and approved the manuscript.

## Conflict of Interest

The authors declare that the research was conducted in the absence of any commercial or financial relationships that could be construed as a potential conflict of interest.

## Publisher’s Note

All claims expressed in this article are solely those of the authors and do not necessarily represent those of their affiliated organizations, or those of the publisher, the editors and the reviewers. Any product that may be evaluated in this article, or claim that may be made by its manufacturer, is not guaranteed or endorsed by the publisher.
