# Supplementary material for: Effect of Environmental Stress on the Nutrient Stoichiometry of the Clonal Plant Phragmites australis in Inland Riparian Wetlands of Northwest China
Source: Front Plant Sci. 2021 Aug 19;12:705319. doi: 10.3389/fpls.2021.705319 (PMC8416684; doi:10.3389/fpls.2021.705319)
Supplement: Supplementary file 1 [file Table_1.DOCX]

**Supplementary Table S1**

SMA analysis of C、N、P stoichiometry in the whole plant of *P. australis*

| log Y vs log X | Habitat | b | 95%CI | p | R^2^ |
| --- | --- | --- | --- | --- | --- |
| C-N | Wetland | **-0.288** | -0.131~-0.635 | <0.01 | 0.726 |
|  | Salt marsh | **0.379** | 0.170~0.845 | <0.05 | 0.562 |
|  | Desert | **0.313** | 0.140~0.699 | <0.01 | 0.676 |
| C-P | Wetland | **-0.254** | -0.123~-0.527 | <0.001 | 0.811 |
|  | Salt marsh | **0.343** | 0.192~0.612 | <0.01 | 0.779 |
|  | Desert | **0.270** | 0.137~0.530 | <0.001 | 0.820 |
| N-P | Wetland | 0.883 | 0.479~1.626 | 0.662 | 0.028 |
|  | Salt marsh | 0.906 | 0.410~2.001 | 0.798 | 0.010 |
|  | Desert | -0.861 | -0.428~-1.734 | 0.655 | 0.030 |
| C:N-P | Wetland | -1.536 | -0.742~-3.181 | 0.229 | 0.199 |
|  | Salt marsh | 0.774 | 0.349~1.713 | 0.509 | 0.065 |
|  | Desert | 1.409 | 0.647~3.064 | 0.368 | 0.117 |
| C:P-N | Wetland | -1.869 | -0.839~-4.160 | 0.119 | 0.310 |
|  | Salt marsh | -1.086 | -0.508~-2.327 | 0.821 | 0.008 |
|  | Desert | 1.714 | 0.830~3.538 | 0.134 | 0.289 |
| N:P-C | Wetland | **0.195** | 0.089~0.425 | <0.001 | 0.868 |
|  | Salt marsh | **-0.250** | -0.130~-0.478 | <0.001 | 0.854 |
|  | Desert | **-0.136** | -0.061~-0.300 | <0.001 | 0.931 |
