# Supplementary material for: Effect of Environmental Stress on the Nutrient Stoichiometry of the Clonal Plant Phragmites australis in Inland Riparian Wetlands of Northwest China
Source: Front Plant Sci. 2021 Aug 19;12:705319. doi: 10.3389/fpls.2021.705319 (PMC8416684; doi:10.3389/fpls.2021.705319)
Supplement: Supplementary file 2 [file Table_2.DOCX]

**Supplementary Table S2**

SMA analysis of C, N, P stoichiometry in leaf of *P. australis*

| log Y vs log X | Habitat | b | 95%CI | p | R^2^ |
| --- | --- | --- | --- | --- | --- |
| C-N | Wetland | **-0.217** | -0.105~-0.450 | <0.001 | 0.859 |
|  | Salt marsh | **-0.333** | -0.149~-0.741 | <0.01 | 0.643 |
|  | Desert | 0.143 | -0.251~-1.239 | 0.143 | 0.279 |
| C-P | Wetland | **-0.171** | -0.078~-0.375 | <0.001 | 0.895 |
|  | Salt marsh | **0.334** | 0.172~0.650 | <0.01 | 0.734 |
|  | Desert | **0.192** | 0.087~ 0.426 | <0.001 | 0.865 |
| N-P | Wetland | 0.790 | 0.488~1.278 | 0.297 | 0.153 |
|  | Salt marsh | -1.003 | -0.450~-2.236 | 0.993 | 0.003 |
|  | Desert | **-0.345** | -0.161~-0.739 | <0.01 | 0.649 |
| C:N-P | Wetland | -0.882 | -0.523~-1.488 | 0.603 | 0.041 |
|  | Salt marsh | 1.091 | 0.500~2.379 | 0.818 | 0.008 |
|  | Desert | **0.416** | 0.195~0.888 | <0.05 | 0.531 |
| C:P-N | Wetland | -1.337 | -0.859~-2.081 | 0.171 | 0.249 |
|  | Salt marsh | 0.840 | 0.377~1.876 | 0.657 | 0.030 |
|  | Desert | 2.859 | 1.328~6.159 | <0.01 | 0.640 |
| N:P-C | Wetland | **-0.305** | -0.139~-0.673 | <0.01 | 0.697 |
|  | Salt marsh | **-0.226** | -0.110~-0.464 | <0.001 | 0.851 |
|  | Desert | **-0.165** | -0.0747~-0.365 | <0.001 | 0.899 |
