# Supplementary material for: Effect of Environmental Stress on the Nutrient Stoichiometry of the Clonal Plant Phragmites australis in Inland Riparian Wetlands of Northwest China
Source: Front Plant Sci. 2021 Aug 19;12:705319. doi: 10.3389/fpls.2021.705319 (PMC8416684; doi:10.3389/fpls.2021.705319)
Supplement: Supplementary file 3 [file Table_3.DOCX]

**Supplementary Table S3**

SMA analysis of C, N, P stoichiometry in stem of *P. australis*

| log Y vs log X | Habitat | b | 95%CI | p | R^2^ |
| --- | --- | --- | --- | --- | --- |
| C-N | Wetland | **0.227** | 0.116~0.443 | <0.001 | 0.871 |
|  | Salt marsh | **0.290** | 0.141~0.595 | <0.01 | 0.765 |
|  | Desert | **0.208** | 0.094~0.460 | <0.001 | 0.846 |
| C-P | Wetland | **0.404** | 0.186~0.875 | <0.05 | 0.542 |
|  | Salt marsh | **0.441** | 0.213~0.913 | <0.05 | 0.514 |
|  | Desert | **-0.205** | -0.092~-0.455 | <0.001 | 0.847 |
| N-P | Wetland | 1.779 | 0.808~3.919 | 0.144 | 0.279 |
|  | Salt marsh | 1.523 | 0.773~3.000 | 0.204 | 0.219 |
|  | Desert | -0.982 | -0.457~-2.108 | 0.860 | 0.007 |
| C:N-P | Wetland | -1.574 | -0.710~-3.489 | 0.249 | 0.184 |
|  | Salt marsh | -1.366 | -0.668~-2.793 | 0.367 | 0.117 |
|  | Desert | 0.963 | 0.446~2.077 | 0.918 | 0.002 |
| C:P-N | Wetland | 0.539 | 0.241~1.204 | 0.125 | 0.303 |
|  | Salt marsh | -0.583 | -0.277~-1.228 | 0.145 | 0.278 |
|  | Desert | 1.064 | 0.498~2.270 | 0.866 | 0.004 |
| N:P-C | Wetland | **0.218** | 0.103~0.457 | <0.001 | 0.851 |
|  | Salt marsh | **0.352** | 0.160~0.775 | <0.05 | 0.619 |
|  | Desert | **0.126** | 0.057~0.277 | <0.001 | 0.940 |
