# Supplementary material for: Effect of Environmental Stress on the Nutrient Stoichiometry of the Clonal Plant Phragmites australis in Inland Riparian Wetlands of Northwest China
Source: Front Plant Sci. 2021 Aug 19;12:705319. doi: 10.3389/fpls.2021.705319 (PMC8416684; doi:10.3389/fpls.2021.705319)
Supplement: Supplementary file 4 [file Table_4.DOCX]

**Supplementary Table S4**

SMA analysis of C, N, P stoichiometry in rhizome of *P. australis*

| log Y vs log X | Habitat | b | 95%CI | p | R^2^ |
| --- | --- | --- | --- | --- | --- |
| C-N | Wetland | **-0.281** | -0.126~-0.627 | <0.01 | 0.729 |
|  | Salt marsh | **-0.136** | -0.061~-0.301 | <0.001 | 0.931 |
|  | Desert | **0.133** | 0.061~0.289 | <0.001 | 0.937 |
| C-P | Wetland | **-0.230** | -0.104~-0.509 | <0.01 | 0.814 |
|  | Salt marsh | **0.067** | 0.030~0.149 | <0.001 | 0.983 |
|  | Desert | **-0.120** | -0.054~-0.269 | <0.001 | 0.944 |
| N-P | Wetland | -0.819 | -0.369~-1.817 | 0.607 | 0.040 |
|  | Salt marsh | -0.496 | -0.224~-1.098 | 0.081 | 0.373 |
|  | Desert | 0.906 | 0.439~1.870 | 0.777 | 0.012 |
| C:N-P | Wetland | 0.850 | 0.382~1.895 | 0.678 | 0.026 |
|  | Salt marsh | 0.512 | 0.232~1.131 | 0.094 | 0.350 |
|  | Desert | 0.906 | 0.439~1.870 | 0.714 | 0.020 |
| C:P-N | Wetland | 1.298 | 0.584~2.882 | 0.505 | 0.066 |
|  | Salt marsh | 1.996 | 0.901~4.422 | 0.085 | 0.365 |
|  | Desert | -1.120 | -0.535~-2.343 | 0.749 | 0.016 |
| N:P-C | Wetland | **0.167** | 0.0749~0.371 | <0.001 | 0.896 |
|  | Salt marsh | **-0.057** | -0.026~-0.125 | <0.001 | 0.988 |
|  | Desert | **0.122** | 0.056~0.263 | <0.001 | 0.948 |
