# Supplementary material for: Effect of Environmental Stress on the Nutrient Stoichiometry of the Clonal Plant Phragmites australis in Inland Riparian Wetlands of Northwest China
Source: Front Plant Sci. 2021 Aug 19;12:705319. doi: 10.3389/fpls.2021.705319 (PMC8416684; doi:10.3389/fpls.2021.705319)
Supplement: Supplementary file 5 [file Table_5.DOCX]

**Supplementary Table S5**

SMA analysis of C, N, P stoichiometry in root of *P. australis*

| log Y vs log X | Habitat | b | 95%CI | p | R^2^ |
| --- | --- | --- | --- | --- | --- |
| C-N | Wetland | **-0.360** | -0.161~-0.804 | <0.05 | 0.595 |
|  | Salt marsh | **0.188** | 0.088~0.403 | <0.001 | 0.882 |
|  | Desert | **0.229** | 0.103~0.512 | <0.01 | 0.811 |
| C-P | Wetland | **-0.303** | -0.176~-0.522 | <0.001 | 0.847 |
|  | Salt marsh | **-0.132** | -0.062~-0.282 | <0.001 | 0.941 |
|  | Desert | **0.284** | 0.128~0.632 | <0.01 | 0.727 |
| N-P | Wetland | 0.843 | 0.381~1.866 | 0.658 | 0.030 |
|  | Salt marsh | 0.700 | 0.329~1.488 | 0.333 | 0.134 |
|  | Desert | -1.238 | -0.569~-2.691 | 0.572 | 0.048 |
| C:N-P | Wetland | -0.899 | -0.428~-1.889 | 0.766 | 0.014 |
|  | Salt marsh | -0.665 | -0.322~-1.373 | 0.250 | 0.184 |
|  | Desert | 1.259 | 0.582~2.725 | 0.539 | 0.056 |
| C:P-N | Wetland | -1.481 | -0.667~-3.289 | 0.317 | 0.142 |
|  | Salt marsh | -1.505 | -0.697~-3.251 | 0.279 | 0.164 |
|  | Desert | 0.815 | 0.375~1.769 | 0.586 | 0.045 |
| N:P-C | Wetland | **0.254** | 0.133~0.483 | <0.001 | 0.851 |
|  | Salt marsh | **0.134** | 0.070~0.259 | <0.001 | 0.956 |
|  | Desert | **-0.158** | -0.071~-0.352 | <0.001 | 0.905 |
